# Cytotoxicity Enhancement in Breast Cancer Cells with Carbonate Apatite-Facilitated Intracellular Delivery of Anti-Cancer Drugs

**DOI:** 10.3390/toxics6010012

**Published:** 2018-02-05

**Authors:** Tahereh Fatemian, Ezharul Hoque Chowdhury

**Affiliations:** Jeffrey Cheah School of Medicine and Health Sciences, Monash University Malaysia, Jalan Lagoon Selatan, 46150 Bandar Sunway, Selangor, Malaysia; tfat1@student.monash.edu

**Keywords:** carbonate apatite nanoparticles (NPs), paclitaxel, cytotoxicity, breast cancer, tumour regression

## Abstract

Pharmacotherapy as the mainstay in the management of breast cancer has demonstrated various drawbacks, including non-targeted bio distribution and narrow therapeutic and safety windows. Thus, enhancements in pharmacodynamic and pharmacokinetic profiles of the classical anti-cancer drugs could lead to improved efficacy against cancer cells. Therefore, inorganic pH-dependent carbonate apatite (CA) nanoparticles were utilized to efficiently deliver various drugs into cancer cells. Following characterization and various modifications in the structure of CA complexes with different drugs, lifted outcomes were achieved. Markedly, complexing paclitaxel with CA resulted in 20.71 ± 4.34% loading efficiency together with 24.14 ± 2.21% enhancement in cytotoxicity on MCF-7 cells plus superior in vivo anti-tumour efficacy compared to free paclitaxel.

## 1. Introduction

Breast cancer is the most frequently diagnosed cancer in women and the leading cause of cancer death among women. In spite of enhanced preventive measures and treatment strategies, the risk of relapse still remains as high as 20–30% with 5–7% of breast cancer patients developing metastatic disease later on in their lives [1,2]. Currently, breast cancer management is a combination of surgery, radiation, and pharmacotherapy with several drawbacks to each and every modality. Conventional chemotherapy as the mainstay, hormonal therapy, targeted therapy, and immunotherapy, namely recombinant proteins and monoclonal antibodies, and also radiotherapy are all inadequate due to major drawbacks [3,4]. Commonly, non-specificity and systemic toxicity are the underlying reasons for unwanted effects of chemotherapy on normal cells and, possibly, subsequent limited therapeutic effect and increased morbidity [5,6,7]. The next downside is the resistance of cancer cells to apoptosis and to cytotoxic agents accounting for 90% of treatment failure [8]. Thus, the need for a new discipline in breast cancer therapy, manoeuvring the pharmacodynamic and pharmacokinetic profile of classical anti-cancer drugs for improved cytotoxic efficacy against cancer cells, is strongly highlighted [9,10]. 

As the most active cytotoxic drug class, taxanes are the standard of care in early and metastatic breast cancer and the first generation, docetaxel and paclitaxel, are among the most efficient agents and the most commonly used in all stages of breast cancer [2,11]. Paclitaxel is a natural compound from which docetaxel is semi-synthetically obtained with higher water solubility. Interestingly, with different response rates, distinct mechanisms of action are assumed for paclitaxel and docetaxel, leaving docetaxel with higher anti-tumour potency. Thus, some patients who do not respond to paclitaxel still respond to docetaxel [1,11]. The mechanism of action of docetaxel and paclitaxel involves binding to free tubulin and generating stable microtubules, while preventing disassembly at the same time. These abnormal microtubule clusters inhibit cell division by cell cycle arrest at the G2/M phase, leading to apoptosis. Docetaxel may also suppress the antiapoptotic gene *Bcl2* and induce the cell cycle inhibitor p27 [11,12]. Despite all the benefits, it has been shown that with classical formulations, 95% of the drug is delivered to normal tissue which means that only 2–5% reaches the cancer cells [12] with eventual significant systematic toxicity. Additionally, the low solubility profile in aqueous solutions, commonly within the range of mg/mL, necessitates the use of synthetic vehicles (polyethoxylated castor oil for paclitaxel and polysorbate-ethanol 80 for docetaxel) in the formulation which would contribute highly to the toxic effects [12]. Mitomycin C is an antitumour antibiotic discovered in cultures of *Streptomyces caespitosus* with extensive clinical use. As an alkylating agent, mitomycin C crosslinks the complementary strands of the DNA double helix [13]. Considering the hypoxic condition within a solid tumour and mitomycin’s higher potency in hypoxia, the drug would be considerably beneficial in cancer local therapy. However, due to acute and chronic toxicities, such as irreversible myelosuppression and hemolyticuremic syndrome, new strategies aiming for better safety profile and efficacy are required [14].

To address the above mentioned problems and to optimize the functions of anti-cancer drugs, nano-vectors could be utilized, conferring the advantages of improved water solubility without the need for toxic solvents, appropriate size, biocompatibility, biodegradability, and optimized pharmacokinetics and pharmacodynamics of the incorporated drug [3,12]. Nanotechnology tools in drug delivery include micelles, liposomes, and polymers, namely poly(ethylene glycol), steroids, peptides, hyaluronic acid, folate, fatty acids, antigens, dendrimers, oligomers, like chitosan and cyclodextrin, nanotubes, and various types of nanoparticles (NPs). Vectorization can be passive with weak drug–vector bonds assembling the drug or active through covalent drug–vector bonds that are cleaved at the target site [12,15]. The obstacle in incorporating hydrophobic drugs into carriers is the low affinity and quick release of the drug which could be solved by formulating hydrophilic prodrugs, such as drug-hydrocarbon anchor conjugates, before loading the drugs to the carriers [16]. NAB paclitaxel, marketed as Abraxane, utilizes nanoparticles loaded with paclitaxel coated by human serum albumin as an organic vehicle to improve endothelial transcytosis. The modification has enhanced tumour penetration, plasma clearance, and volume of distribution. Thus, the tolerated dose is higher than that of the castor oil formulation, leading to increased efficiency [12,17]. Aiming for elevated exposure time and local drug concentration, mitomycin C has been formulated with colloidal delivery systems and also nano- or microparticles consisting of polymers, such as albumin, dextran, estradiol, N-succinyl-chitosan, hydrogels, polybutylcyanoacrylate, and poly-epsilon-caprolactone. However, the low stability of these loaded vectors after local administration in vivo prevents their superior performance in drug delivery [14,18].

With the recent surge in nanomaterial synthesis, nano-oncology utilizes nanomaterials with the size of a few nanometers to 1000 nm to treat human malignancies at the molecular level. Most nanoparticles can be classified into two major types: organic materials, such as liposomes, dendrimers, carbon nanotubes, emulsions, and other polymers, and inorganic elements, which usually have metals as a core with a protective organic coating [17,19,20]. Nanocarriers are designed with various properties based on their physicochemical characteristics, such as size distribution, surface morphology, steric stabilization, loading efficiency, release kinetics, and hemodynamic properties. The targeting process through nanocarriers is either passive or active. In passive targeting particle transportation into the tumour cells and interstitium is achieved through leaky tumour capillary openings. Fast angiogenesis due to the high consumption of oxygen causes leaky vasculature and weak lymphatic circulation in tumoural tissue, resulting in the enhanced permeability and retention effect (EPR effect). Conversely, active delivery involves targeting specific sites by addition of different moieties [3,11]. Based on the versatile specifications of nanoparticles and also the characteristics of the loaded agent, they may be employed to deliver various types of therapeutics, such as small molecule drugs, peptides, proteins, oligo- and polynucleotides, and genes [15]. 

Inorganic carbonate apatite (CA) is a recently-developed nano-carrier synthesized via calcium phosphate precipitation in the presence of bicarbonate with controlled crystal growth, generating particles between 50 and 300 nm. Therefore, this new delivery vehicle could be ideal in the context of efficient endocytosis, fast dissolution rate in endosomal acidic pH, and effective release of loaded therapeutics. With the molecular formula of Ca_10_·(PO_4_)_6 − X_·(CO_3_)_X_·(OH)_2_ particles are formed through a flexible process by incubating a bicarbonate-buffered medium containing appropriate concentrations of phosphate and calcium salts in presence of a therapeutic agent. The required chemical reaction takes place with development of solution supersaturation with respect to Ca^2+^, PO_4_^3−^ and HCO_3_^−^, and the therapeutic agent is electrostatically associated either with the cationic (Ca^2+^-rich) domains or anionic sites (PO_4_^3−^ or HCO_3_^−^ -rich) of the particles depending on their net charges [21,22,23,24,25]. The structural role of bicarbonate, though to a minor amount, is to control the size of the particles and inhibit aggregation [22]. In addition, high acid solubility as a result of carbonate substitution would provide the feature of fast endosomal escape for the loaded materials. With dissolution of endocytosed apatite particles in endosomes, the released Ca^2+^ in cytoplasm could be processed by ATP-driven Ca^2+^ pumps and the Na^+^/Ca^2+^ exchanger [26,27]. Therefore, with rapid degradation of CA particles and no indication of toxicity, these materials appear to be clinically safe.

## 2. Methods and Materials

### 2.1. Materials

Dulbecco’s modified eagle medium (DMEM), calcium chloride dehydrate (CaCl_2_·2H_2_O), sodium bicarbonate (NaHCO_3_), dimethyl sulphoxide (DMSO), thiazolyl blue tetrazolium bromide (MTT), and ethylene diamine tetraacetic acid (EDTA) were purchased from Sigma-Aldrich (St. Louis, MO, USA). DMEM powder, fetal bovine serum (FBS), trypsin-ethylenediamine tetraacetate (trypsin-EDTA), and penicillin-streptomycin were obtained from Gibco BRL (CA, USA). Anticancer drugs including paclitaxel (Pac), docetaxel (Doc), mitomycin C from streptomyces caespitosus (Mito) and topotecan hydrochloride hydrate (Topo) were purchased from Sigma. Topotecan is not approved for breast cancer and is used as a control drug in this study to observe cytotoxic effects of variously indicated medications against breast cancer cells. Acetonitrile (ACN) and triethylamine (TEA) were from Fisher Scientific (Loughborough, UK). All the chemicals used for HPLC were HPLC grade. MCF-7 and 4T1 cells were originally from ATCC.

### 2.2. Measurement of Particle Size

Size of the NPs formed in different concentrations of CaCl_2_ were measured using Zeta Sizer (Nano ZS, Malvern) after adding 10% FBS and temporarily keeping on ice to prevent particle aggregation. A refractive index ratio of 1.325 was set for the estimation of particle diameter. Data were analysed using Zetasizer software 6.20 and all samples were measured in duplicate.

### 2.3. Turbidity Measurement

As an indirect method to investigate the precipitation following nucleation in a supersaturated solution, turbidity determination has been employed, interpreting time-dependent changes in optical density in terms of particle formation or growth. Thus, optical density of the particle suspension at 320 nm against a blank has been linked to the amount of nanoparticles synthesized. This method involves media preparation and addition of all reactants followed by 30 min incubation at 37 °C for 30 min, as mentioned earlier and the optical density measurement. The reason for selecting 320 nm as the particular wavelength for turbidity measurement of particle’s suspension is the least overlap with background components of the sample at this wavelength. All spectroscopic readings were blank-corrected, so that only the value for the apatite present in the sample is concluded (performed by a Shimadzu UV-1800 UV spectrophotometer). 

### 2.4. Cell Culture

MCF7 and 4T1 cells obtained from ATCC were cultured in Dulbecco’s modified Eagle’s medium (DMEM, Gibco) supplemented with 10% fetal bovine serum (FBS), 1% penicillin and streptomycin, and 1% HEPES (4-(2-hydroxyethyl)-1-piperazineethanesulfonic acid) at 37 °C in a humidified incubator in 5% CO2.

### 2.5. Generation of CA Particles

Bicarbonated DMEM medium prepared by dissolving DMEM powder and the addition of 44 mM sodium bicarbonate in Milli-Q (double-distilled) water was used to prepare CA nanoparticles. Following pH adjustment to 7.5, 1 to 9 mM of exogenous Ca^2+^ from 1 mM CaCl_2_ was properly mixed prior to 30 min incubation at 37 °C and turbidity measurement at 320 nm against fresh medium as a blank. 

### 2.6. Effect of Ca^2+^, PO_4_^3−^ (Pi), Drug, and pH on Crystal Growth Kinetics

Different pHs, as well as various Ca and/or Pi concentrations, with the fixed ratio of Ca:Pi equal to 10:6 (according to the molecular formula of carbonate apatite Ca_10_·(PO_4_)_6-X_·(CO_3_)_X_·(OH)_2_), in he presence of different concentrations of anticancer drugs, were investigated. Both 1 mM CaCl_2_ stock and 0.5 mM Na_2_HPO_4_·7H_2_O stock solutions were used. The pH of the HCO_3_^−^-buffered medium was set to 6.5, 7.5, and 8.5, the reactants were mixed in order, and at required concentrations based on the experimental design. Spectroscopic reading at 320 nm was performed for monitoring the changes in the formation and growth of differently-formulated particles.

### 2.7. Calculating Drug Encapsulation Efficiency

The drug encapsulation efficiency of CA NPs was analysed by a high-performance liquid chromatography (HPLC) system with a HC-C18 analytical column (250 mm × 4.6 mm, 5 μm, C18, Agilent Technologies, Santa Clara, CA, USA). To detect and quantify the drug bound to nanoparticles, the samples were formulated as mentioned earlier by applying 7 mM calcium and 100 µM of each drug. The particles were spun down and the amount of drug in the pellet and supernatant was analysed to track the presence of drug in both free and nanoparticle-bound forms. The pellet was treated with ethylenediaminetetraacetic acid (EDTA) to dissolve the particles in order to obtain the particle-bound drugs. Free media, free drug in media, and nanoparticles in media were also analysed for background elimination. The quantification of the available drug in each sample was performed based on the standard curves obtained with analysing 20, 40, 60, 80, and 100 µM of each drug.

The details of HPLC method for each drug are summarized in Table 1

The drug encapsulation efficiency was calculated based on the following formula:

$$\mathrm{Interaction}\;\mathrm{efficiency}\;\left(\%\right)=\frac{{\left[X\right]}_{\mathrm{CA}/\mathrm{drug}}-{\left[X\right]}_{\mathrm{free}\;\mathrm{drug}}}{{\left[X\right]}_{\mathrm{initial}}}\times 100$$

where [X]_free drug_ and [X]_CA/drug_ are the amount of drug present in the pellets (calculated form the standard curves) following centrifugation of the free drug and NP-drug samples, respectively, and [X] _initial_ is the amount of drug initially used for preparation of the samples to perform HPLC. Each experiment was done in triplicate and the results are shown as the mean ± SD.

### 2.8. In Vitro Cytotoxicty of NPs

The effect of CA particles formulated with different calcium concentrations on cell viability in MCF-7 cells was assessed by MTT assay. The cells were seeded on 24-well plates with a density of 5 × 10^4^ cells/mL. Following 24 h incubation, cells were treated with 1 mL of media only as a control or 1 mL of different apatite samples. CA NPs were formed with 1–7 mM Ca^2+^ and 10% FBS was added to all samples and controls after incubation to stop further particle formation. After 44 h of treatment of the cells, cell viability was measured by 3-(4,5-dimethylthiazol-2-yl)-2,5-diphenyltetrazolium bromide (MTT) assay. 

### 2.9. In Vitro Cytotoxicity of NPs/Drug

To prepare the complexes of NPs plus drugs, different concentrations of drugs and CaCl_2_ were mixed with 1 mL of fresh serum-free HCO_3_^−^-buffered medium (pH 7.4), followed by incubation for 30 min at 37 °C for complete generation of drug/CA particles. Rinsed cells, which had been plated one day before at 5 × 10^4^ cell/mL, were treated with 1 mL of medium with the generated drug-containing particles plus 10% FBS. Media only, different free drug samples, and nanoparticles only were used as controls. For MCF-7 cells, 1 nM to 1 µM of each drug was used and apatite was formed with 3 mM CaCl_2_ for Doc and Mito and 4 mM CaCl2 for Pac and Topo. 4T1 Cells were treated with 1 nM to 1 µM of Doc and 10 nM to 10 µM of Pac, Mito, and Topo in the form of free drugor apatite/drug. In apatite preparation 3 mM CaCl_2_ was used for Doc and 4 mM CaCl_2_ for Pac and Mito and Topo. On the fourth day of the treatment protocol, MTT assay was performed to measure the cell viability.

### 2.10. Effect of Particle Synthesis Process on Cytotoxicity

The effect of pH and exogenously added Ca^2+^ as well as PO_4_^3−^ within the fixed ratio of Ca:Pi (being equal to 10:6), on the synthesis of particles as evaluated by the subsequent particle-mediated cytotoxicity was studied on MCF7 and 4T1. Apatite/drug was formed with 100 nM of drugs in bicarbonated media with three different pHs (6.5, 7.5, and 8.5). Various concentrations of total exogenous and endogenous calcium and phosphate were added in 10:6 ratios for apatite formation. The cells were treated with media or free drug or apatite/drug for each pH and Ca/Pi concentration. Plating was done with 5 × 10^5^ cell/well one day before treatment. MCF-7 cells received Pac, Mito, and Topo, whereas in 4T1 treatment Doc was also included. Two days after treatment, the effect of the particle synthesis process on cytotoxicty was studied by means of cell viability analysis and MTT assay.

### 2.11. Cell Viability Assay by MTT

MCF-7 and 4T1 cells were cultured in a 75 cm^2^ tissue culture flasks (Nunc, Orlando, FL, USA) and maintained in the DMEM media (pH 7.4) supplemented with 10% FBS, and penicillin and streptomycin antibiotics, in a CO_2_ incubator. Approximately 5 × 10^4^ cells were seeded on 24-well plates (Griener, Frickenhausen, Germany). After 24 h, the cells were treated with media (untreated), NPs and drug-loaded NPs for two consecutive days. Lastly, 50 µL of MTT (5 mg/mL in PBS) was added to each well so that metabolically-active cells could form formazan crystals, which were subsequently (after 4 h) dissolved by adding 300 µL of dimethyl sulfoxide (DMSO). The plates were then agitated on the built-in plate shaker for 20 s. Optical density (OD) at 595 mm wavelength with a 630 nm reference wavelength was measured using a multi-well plate reader (microplate spectrophotometer, Biorad) for formazan quantification, and the percentage of metabolically-active cells (cell viability, CV) was calculated for treated samples using the following equation:

$$\%\;\mathrm{of}\;\mathrm{cell}\;\mathrm{viability}\;(\mathrm{CV})=\frac{{\mathrm{OD}}_{\mathrm{treated}}-{\mathrm{OD}}_{\mathrm{reference}}}{{\mathrm{OD}}_{\mathrm{untreated}}-{\mathrm{OD}}_{\mathrm{reference}}}\times 100$$

Each experiment was done in triplicate and expressed in graphs as the mean ± SD of the percentage of cell viability. All the experiments were repeated two to three times. 

Accordingly, cytotoxicity enhancement (%) due to drug entrapment in NPs was calculated using the following formula

$$\%\;\mathrm{Enhancement}\;\mathrm{in}\;\mathrm{cytotoxicity}\;(\%)={\mathrm{CV}}_{\mathrm{free}\;\mathrm{drug}}-{\mathrm{CV}}_{\mathrm{NP}/\mathrm{drug}}$$

### 2.12. Formulation of Particles for In Vivo Study

Nanoparticles were formed by mixing of CaCl_2_ in 100 μL of freshly-prepared bicarbonated (44 mM) DMEM media and then 30 min incubation at 37 °C. Samples were maintained on ice to prevent aggregation during injection. For complexing with drugs 1.25 mg/Kg of Pac and 1 mg/kg of Doc were used before incubation. The resulting therapeutics were used for intravenous treatment of animals. 

### 2.13. 4T1 Induced Breast Cancer Murine Model

Female BALB/c mice (6–8 weeks old) weighing 15–20 g were maintained in a 12:12 light:dark condition and provided with food ad libitum and water. All experiments were done in accordance with the regulations imposed by Monash University Animal Welfare Committee. The animal use protocol was approved by Monash Animal Ethics. 4T1 cells (in 100 μL PBS) were injected subcutaneously on the mammary fat pad of mice (considered as day 1) and the mice were checked regularly for the development of tumours by touching the area of injection with an index finger. When the volume of the tumour reached an average of 13.20 ± 2.51 mm^3^, mice were randomized into different groups and treated intravenously via the right or left caudal vein. The mice were closely monitored and their body weights and tumour outgrowth were recorded every other day for a period of 30 days. The tumour volume was calculated using the following formula:

$$\mathrm{Tumor}\;\mathrm{volume}\;\left({\mathrm{mm}}^{3}\right)=\frac{\left(\mathrm{Length}\times {\mathrm{Width}}^{2}\right)}{2}$$

## 3. Results and Discussion

### 3.1. Generation of Carbonate Apatite Particles

Synthesis of carbonate apatite particles was confirmed by measuring the turbidity of the prepared samples and also the changes in the particle formation with increasing calcium concentration were observed.

**Figure 1:**
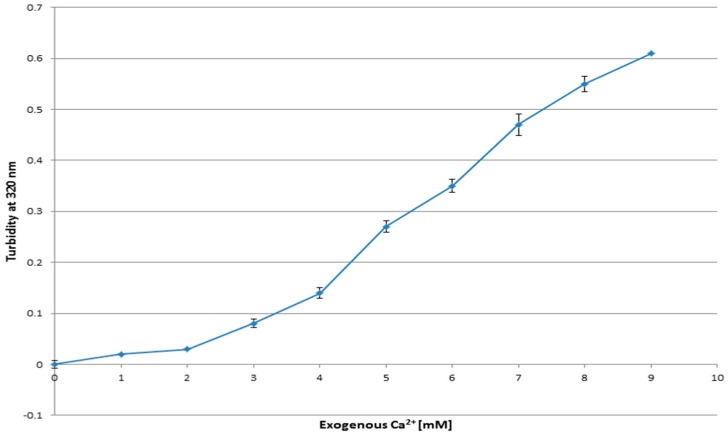
Apatite was formed by addition of 1 to 9 µL of exogenous Ca^2+^ from 1 mM CaCl_2_ to bicarbonated DMEM (pH 7.5). After 30 min of incubation at 37 °C turbidity was measured at 320 nm against fresh media as a blank. The experiment was perfomed in duplicate and data are presented as the mean ± SD.

Increasing optical density with escalating Ca^2+^ concentrations (1 to 9 mM) in HCO_3_^−^-buffered DMEM (pH 7.5) could be linked to the rise in particle growth, which is proportionate to the calcium increase, as the concentrations of HCO_3_^−^ and PO_4_^3−^ are fixed (Figure 1). With the consumption of the entire PO_4_^3−^ available in the medium, the particle formation possibly reached a steady state.

The basis for the synthesis process of carbonate apatite nanoparticles is the availability of optimal concentrations of three electrolytes, Ca^2+^, PO_4_^3−^, and HCO_3_^−^, creating a supersaturated medium. Co-precipitation of the reactants could also be affected by temperature and pH of the medium; however, the current protocol of maintaining 37 °C and 30 min of incubation was optimized in the earlier study [11]. Any changes in the details of the process could be reflected in the resulting product; for example, the increase in the initial concentrations of the reactants would induce the particle formation by promoting the driving force for the synthesis process and the result could be the production of more carbonate apatite particles and the resulting increased particle size due to the aggregation of the nanoparticles [11].

As a matter of fact, based on Le Châtelier’s Principle and the tendency of each equilibrium to preserve its balance, addition of sufficient concentrations of Ca^2+^ as the common ion with the carbonate apatite formation reaction would decrease the solubility of the ionic precipitate, shifting the equilibrium to the right and causing more precipitation of carbonate apatite.

### 3.2. Size and Zeta Potential of CA/pac

**Figure 2:**
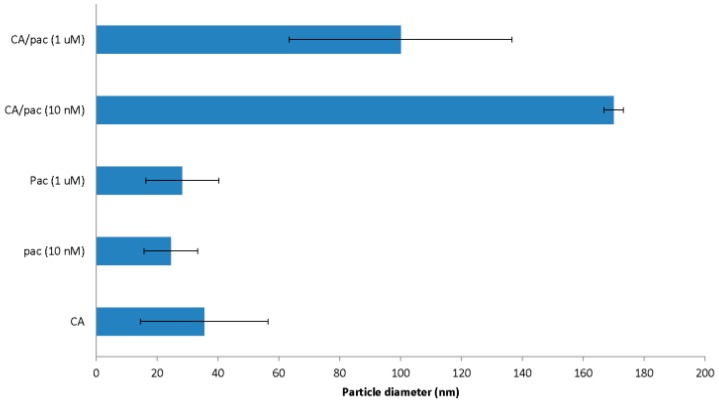
Size measurement for carbonate apatite particle complexes formulated by incorporation of 3 mM of CaCl_2_ together with 10 nM and 1 µM pac. The samples were then incubated at 37 °C for 30 min and also exposed to 10% FBS and maintained on ice prior to size measurement.

**Figure 3:**
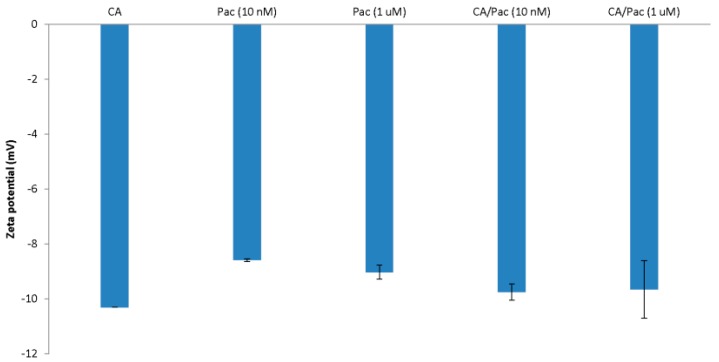
Zeta potential measurement for carbonate apatite particle complexes formulated by incorporation of 3 mM of CaCl_2_ together with 10 nM and 1 uM pac. The samples were then incubated at 37 °C for 30 min and also exposed to 10% FBS and maintained on ice prior to size measurement.

To produce complexes of carbonate apatite and pac for size and zeta potential measurements (Figure 2 and Figure 3), 3 mM of CaCl_2_ plus 10 nM or 1 µM of pac were applied prior to 30 min incubation at 37 °C. Afterwards, the samples were exposed to 10% FBS and incubated on ice to prevent further particle formation and growth.

Size of CA formed with 3 mM of Ca is reported as less than 50 nM. By incorporation of 10 nM pac, NPs reach the maximum size of around 170 nm.

The reason for the relatively large deviation in particle size in presence of higher hydrophobic drug concentrations could be the contact of aqueous and organic phases and the fact that DMSO-soluble drugs might induce particles aggregation via interacting with multiple growing particles in the aqueous phase.

### 3.3. Influencing Crystal Growth Kinetics by Ca^2+^, PO_4_^3−^, Drug, and pH

Two Different Ca concentrations of 5 mM and 6 mM with 1 nM to 10 uM Pac concentrations in a ten-fold serial dilution were used to assess the effect of the increased Ca and drug concentration on turbidity and, thereupon, on the induction of particle formation.

**Figure 4:**
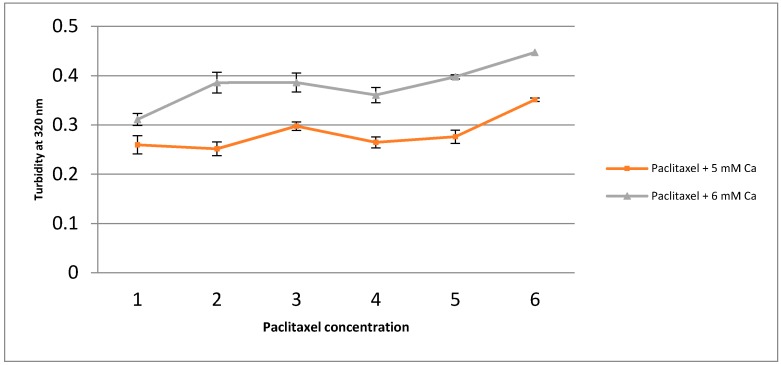
Apatite with or without drugs (0 to 100 µM) were formed in bicarbonated medium with addition of 5 and 6 mM of exogenous calcium chloride in presence or absence of drugs, followed by 30 min incubation at 37 °C. After incubation, turbidity was measured at 320 nm against fresh media as a blank. Values for apatite were calculated by deducting values for the free drug at different concentrations. All experiments were performed in duplicate and data represents the mean ± SD.

As shown in Figure 4, application of 6 mM of Ca^2+^ results in higher overall turbidity as compared to 5 mM Ca^2+^, implying that higher Ca^2+^ concentration is associated with higher particle formation, whereas increasing the paclitaxel amount does not directly contribute to the formation of nanoparticles.

However, the generation of carbonate apatite particles is regulated through promoting or preventing the induction of supersaturation in the bicarbonate-buffered solution containing optimal concentrations of key reacting electrolytes, calcium, phosphate, and, also, drug [28]. Thus, the rate of the chemical reaction between the components of the solution is directly influenced by concentrations of the reactants, pH, and temperature. 

With lower proton concentration at higher pH, a solution’s capacity to accept a proton would increase and, thereafter, the chances of losing a proton for the available molecules would be higher.

Based on the increased ionization of phosphate and bicarbonate, and the development of supersaturation in the solution by increasing the pH of the medium, the incubation temperature, and time, the synthesis process could be pushed towards increased particle formation. Thus, with higher pH or incubation temperature the optimal Ca^2+^ concentrations necessary for the induction of effective particle synthesis are decreased. 

Additionally, concentrations of initially-added Ca^2+^, inorganic phosphate, and HCO_3_^−^ are also directly controlling the process of carbonate apatite formation. The DMEM powder used for the preparation of the medium in which particles are formed contains endogenous phosphate and calcium with 0.9 mM and 1.8 mM concentrations, respectively. 

With the ratio of total endogenous and exogenous Ca and Pi ions fixed as 10:6 (based on the molecular formula and percent composition of calcium and phosphate), the approximate ratio of Ca:Pi equal to 3:1.8, 5:3 and 7:4.2 for the pHs of 6.5, 7.5 and 8.5, respectively, are selected for turbidity measurement experiments.

**Figure 5:**
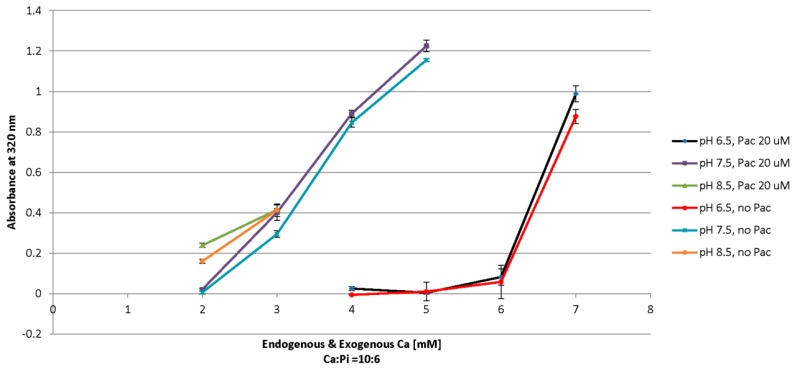
Apatite was formed in bicarbonated medium by mixing with it calcium and Pi at a Ca:Pi ratio of 10: 6 with or without 20 uM Pac at pH 6.5, 7.5, and 8.5. After 30 min of incubation at 37 °C turbidity was measured at 320 nm against fresh media as a blank. Values for apatite were calculated by deducting values for free drug at different concentration. All experiments were perfomed in duplicate and the data represents the mean ± SD.

The rising concentration of calcium and phosphate within the optimized range for each pH (as discussed above) and 20 µM were used to study the effect of pH, Ca, Pi, and drug concentrations on particle formation (Figure 5).

As shown in Figure 5, the difference between optical density of the two lines at each point could be translated as the incorporation of pac into the CA structure, which is mainly achieved at pH 7.5. Slightly higher turbidity for paclitaxel/apatite is observed with 3 to 5 mM calcium in pH 7.5, 2 mM calcium in pH 8.5 and 7 mM calcium in pH 6.5 compared to apatite alone, which could be interpreted as drug interaction with nanoparticles. With the same amount of Pac, 5 mM Ca produces higher turbidity at pH 7.5 than 7 mM Ca at pH 6.5, while with 4 to 6 mM Ca at this pH almost no turbidity is detected. The values are not significantly changed for 2 to 3 mM Ca between pH 8.5 and 7.5 and with 3 mM Ca considerable turbidity of almost 0.4, i.e., substantial particle synthesis, as compared to pH 6.5, is observed.

With increasing concentrations of Pac the turbidity is also slightly augmented, which could be reflecting Pac’s partial incorporation into the particles. The increasing trend of turbidity is almost the same for all three pHs which might reflect the same pattern of pac incorporation. pH 7.5 is showing the highest turbidity, i.e., more particle formation (Figure S1).

### 3.4. Loading Efficiency

Based on the standard curve (Figure S2) and the area under the peak for each sample, the encapsulation efficiency of nanoparticles for docetaxel and paclitaxel was 1.31% and 20.71% (*w*/*w*), respectively. No binding for mitomycin C was observed in this experiment (Table 2).

The significant difference in loading efficiency for different drugs could be explained based on their molecular characteristics. Mitomycin C is a hydrophilic molecule with relatively low molecular weight (334.33), whereas Pac and Doc are both hydrophobic and much larger in size (853.91 and 807.88, respectively). Doc is supposed to be more hydrophilic than pac owing to the tertbutyl carbamate ester in its side chain and a hydroxyl functional group on carbon 10. The ester functional group at C10 in paclitaxel, as the main structural difference, would be protonated and, thus, more electrophilic, resulting in more incorporation with the anionic phosphate/carbonate domains in apatite structure.

### 3.5. Cytotoxicity of Carbonate Apatite on MCF7 Cells

NPs synthesized using 1–7 mM calcium salt do not seem to be toxic to MCF cells. The lowest viability is 97.25 ± 1.62% after treatment with the particles formed with the maximum amount of calcium (7 mM) forming the highest number of NPs (Figure S3).

### 3.6. Cytotoxicity of Drug-Loaded NPs on MCF7 and 4T1

The cytotoxicity of carbonate apatite formulated with drug and free drug were compared on MCF-7 and 4T1 cells.

**Figure 6:**
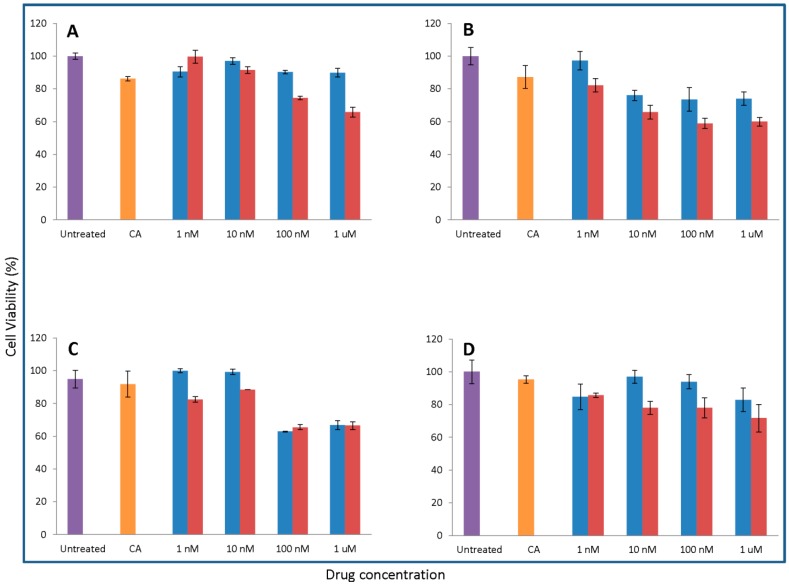
MCF-7 Cells were seeded on 24-well plate at 5 × 10^4^ cell/mL. After 24 h cells were treated with media or free drug or apatite/drug. Apatite was synthesized with 1 nM to 1 µM of the individual drug added to 3 mM CaCl_2_ for Doc (**B**) and Mito (**C**) and 4 mM CaCl_2_ for Pac (**A**) and Topo (**D**). After 44 h of treatment cell viability was measured by MTT assay. Values are calculated as the percentage of cell viability compared to media and apatite as a control for the free drug and apatite/drug, respectively. Data are presented as the mean ± SD. ●Untreated, ●CA treated, ●Free drug treated, ● CA/drug treated.

The cell viability after treatment with apatite/drug is lower than free drug for almost all different concentrations of docetaxel, paclitaxel, and topotecan, which could be due to the enhancement of the uptake of the drug together with nanoparticles through endocytosis, additional to the passive diffusion through the cell membrane. In higher concentrations of mitomycin C, no nanoparticle facilitated uptake by the cells could be assumed, based on the same level of viability in cells treated with free drug and apatite/drug. Notably, the efficacy of carbonate apatite/drug complexes is mostly augmented for 1 μM paclitaxel which exerts 24.14 ± 2.21% enhancement in cytotoxicity resulting from complexation with CA compared to free pac (Figure 6). Interestingly, quantitative assays reveal that cellular uptake of the therapeutics loaded into carbonate apatite is at least three times more efficient compared to the liposome formulation which could further clarify the basis for enhanced efficacy of NP- loaded therapeutics [29]. Additionally, carbonate apatite NPs have been shown to carry electrostatically-associated molecules inside the cells through endocytosis as another non-viral vector and, finally, releasing the bound molecules from the endosome to the cytosol through pH-responsive self-dissolution. Markedly, the intracellular components from the cells treated with loaded and fluorescent labelled NPs showed significant increase in fluorescent intensity compared to those of both untreated and NPs-treated cells [30]. Enhanced uptake of drugs when complexed with carbonate apatite nanoparticles, compared to free drug, based on florescence microscopy and flow cytometry analyses [31], further clarifies the augmented cytotoxicity observed in this study.

**Figure 7:**
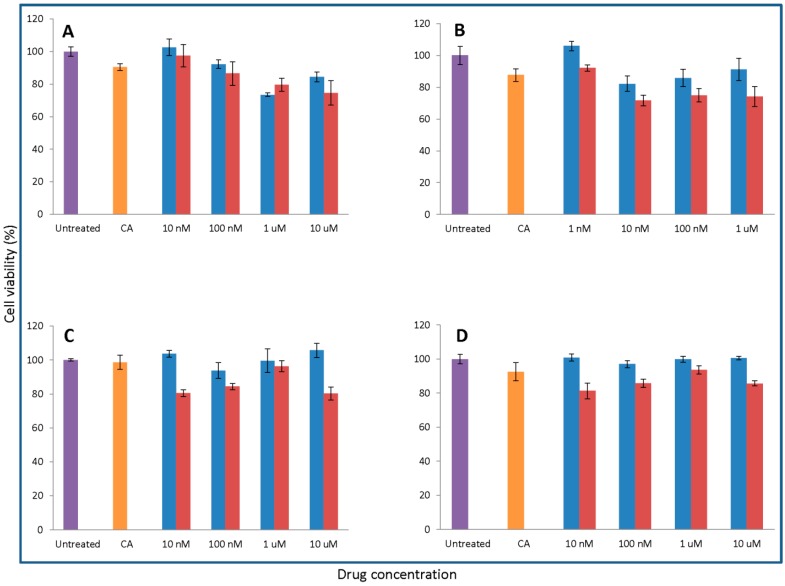
4T1 Cells were seeded on 24-well plates at 5 × 10^4^ cell/mL. After 24 h cells were treated with media or free drug or apatite/drug; 1 nM to 1 µM of Doc (**B**) and 10 nM to 10 µM of Pac (**A**), Mito (**C**), and Topo (**D**) were used for the treatment. Apatite was formed in the presence of the aforementioned drug concentrations using 3 mM CaCl_2_ for Doc and Topo and 4 mM CaCl_2_ for Pac and Mito. After 44 h of treatment cell viability was measured by MTT assay. Values were represented as the percentage of cell viability compared to media and apatite as the control for free drug and apatite/drug, respectively. Data presented as mean ± SD. ●Untreated, ●CA treated, ●Free drug treated, ● CA/drug treated.

As shown in Figure 7, Doc formulated together with apatite, causing the highest reduction in viability of 4T1 cells with 1 µM concentration whereas, for Pac/CA, the most efficient concentration is 10 μM. On 4T1, the maximum enhancement in efficacy equal to 17.04 ± 2.12% is achieved with the application of 1 µM Doc together with CA. Since 4T1 cells were slightly stronger against the anti-cancer drugs used in this study, higher concentrations of drugs were applied in the case of Pac, Mito, and Topo.

Table 3 displays the enhancement in cytotoxic effects of classical anti-cancer drugs delivered via complexation with carbonate apatite compared to the effects of free drug on MCF-7 and 4T1 cells.

### 3.7. Effect of the Synthesis Process on the Cytotoxicity of NPs Formulated with Various Ca and Pi Concentrations at Different pHs on MCF7 and 4T1

A significantly high or low number of particles resulted from the excessive induction or suppression of supersaturation by pH and Ca/Pi concentrations could adversely affect in vitro efficacy in a given volume of cell culture. Thus, effects of these variables on cell viability, which would be affected secondary to changes in the formation of particles, were examined.

**Figure 8:**
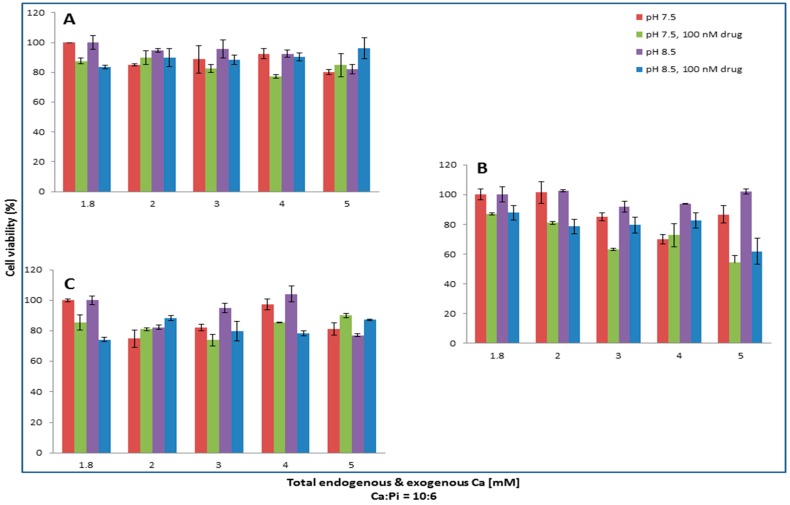
Apatite/drug was formed with 100 nM of Pac (**A**), Mito (**B**), and Topo (**C**) in three different pHs (6.5, 7.5, and 8.5). Various concentrations of total exogenous and endogenous calcium and phosphate were added in 10:6 ratio for apatite formation. MCF-7 cells were seeded one day earlier on 24-well plates at 5 × 10^5^ cell/mL and were treated with apatite alone or apatite/drug. After 44 h of treatment cell viability was measured by MTT assay. Values were represented as the mean percentage of cell viability ± SD as compared to media as control for free drug and apatite only in the case of apatite/drug.

As demonstrated in Figure 8, 100 nM of Pac formulated with 4 mM of calcium and 2.4 mM of phosphate at pH 7.5 is most efficient in killing MCF-7 cells. The increase of pH to 8.5 exerts no positive changes in the cytotoxic effect of CA/pac. The lowest viability is observed with 5 mM Ca plus 3 mM Pi at pH 7.5 for 100 nM Mito, whereas 3 mM calcium in pH 7.5 seems to be the most cytotoxic formulation for Topo/apatite.

**Figure 9:**
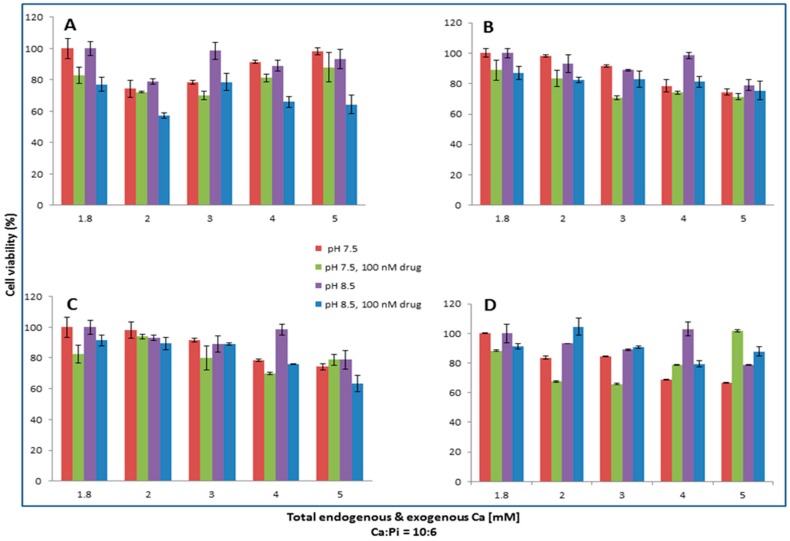
Apatite/drug was formed with 100 nM of Pac (**A**), Doc (**B**), Mito (**C**), and Topo (**D**) in media with three different pHs (6.5, 7.5, and 8.5). Different concentrations of total (exogenous and endogenous) calcium and phosphate were added in a 10:6 ratio for apatite formation. 4T1 cells seeded one day prior to treatment on 24-well plates at 5 × 10^5^ cell/mL were treated with media or free drug or apatite/drug. After 44 h of treatment cell viability was measured by MTT assay. Values were represented as the mean percentage of cell viability ± SD as compared to media as a control for free drug and apatite only in the case of apatite/drug.

Pac complexed with 2 mM of CaCl_2_ at pH 8.5 displays the most enhanced efficacy, while the lowest cytotoxicty for Doc is observed with 3 mM of Ca and 1.8 mM of Pi at pH 7.5. Mito at pH 8.5 and in the presence of 5 mM calcium seems to be more effective against 4T1 cells (Figure 9).

All in all, the observed decline in cytotoxicity with higher Ca^2+^ concentrations could be explained by the fact that the presence of more Ca^2+^ might increase the size of the formed particles, making it more difficult for the cells to internalize the complexes and, thus, decreasing the efficacy. On the other hand, lower concentrations of calcium ions would slow down the synthesis process and also affect the number of the resulting carbonate apatite particles, reflecting the weaker cytotoxic effect. 

### 3.8. In vivo Efficacy of CA/pac

**Figure 10:**
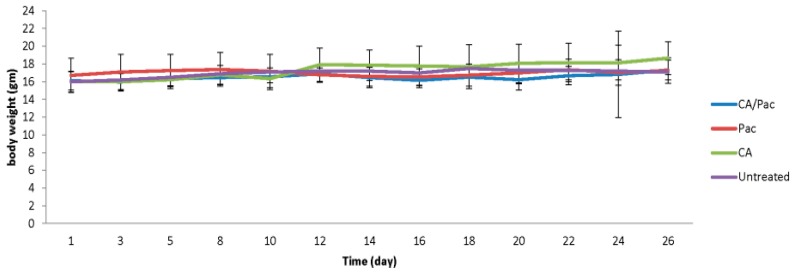

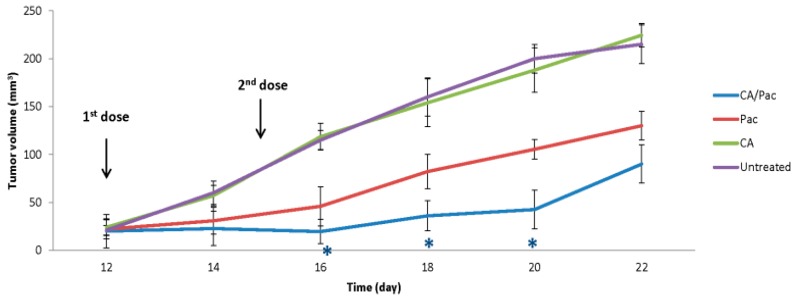
Effect of carbonate apatite particles and particle-bound Pac on a 4T1-induced tumour mouse model. Mice were purchased from the School of Medicine and Health Science animal facility, Monash University, Malaysia. Approximately 10^5^ 4T1 cells were inoculated subcutaneously on the mammary pad of mice on day 1 of the study. Based on tumour volume calculations, mice were randomized and treated intravenously through tail-vein injection on day 12 and 15. The therapeutics included 100 μL of HCO^3^-DMEM containing carbonate apatite formed with 7 μL of 1 M CaCl_2_ with/without 1.25 mg/kg Pac or 1.25 mg/Kg free Pac. Body weight and tumour outgrowth were recorded every other day. Six mice were used per group and data are presented as the mean ± SD of tumour volume. Values are significant with a * *p* value < 0.05 for CA/Pac compared to the free pac group.

The tumour volume and body weight was compared between animals receiving no treatment, carbonate apatite, free pac, or apatite-bound paclitaxel following tumour induction (n = 6) (Figure 10). The data have been presented here as the mean ± SD of body weight and the tumour volume from each group.

According to the results, the pattern of body weight changes with time stayed almost the same for all groups. Thus, the treatment regimens posed nearly no effect on the animals’ body weight. However, based on the t test, the tumour size was significantly reduced in the CA/Pac group on days 16, 18, and 20 compared to the free Pac group. The most significant effect was achieved on day 16 with a *p* value of 0.007.

In conclusion, complexing paclitaxel with carbonate apatite nanoparticles led to augmented outcomes and efficacy against cancer cells both in a cell culture and an animal model. The considerable loading efficiency for CA/Pac could be explained by taking into account the protonation of the ester functional group at C10 in the drug’s structure, making it more electrophilic and promoting its incorporation into anion-rich domains in the apatite structure. Moreover, enhanced in vitro cytotoxicity and significantly superior in vivo efficacy in tumour reduction for CA/Pac compared to free paclitaxel was documented in this study. As revealed by previous quantitative analysis, drug-loaded carbonate apatite shows considerably higher cellular uptake of the drug compared to the free drug (31). Accordingly, endocytosis of NP/drug through the cell membrane could account for the observed enhanced cellular uptake and cytotoxicity. Significantly smaller tumour size in CA/Pac-treated mice at three time points of the in vivo study could hypothetically be related to improved pharmacokinetic or pharmacodynamic profiles of paclitaxel once administered in complexes with carbonate apatite. Thus, extensive animal studies to contemplate bio-distribution patterns and other in vivo characteristics of drug-loaded carbonate apatite are needed for bringing the formulation to clinical trials. 

## Reference

## Figures and Tables

**Table 1:** The drug encapsulation efficiency of carbonate apatite nanoparticles formulated as mentioned earlier by applying 7 mM calcium and 100 µM of each drug.

Drug	Column	Mobile Phase (*v/v*) (all HPLC Grade)	Flow Rate mL/min	Temperature °C	Detection Wavelength (nm)
Paclitaxel	C18 Zorbax, 4.6 × 250 mm	Acetonitrile/Water: 70/30	1	25	227
Docetaxel	C18 Zorbax, 4.6 × 250 mm	Acetonitrile/Water: 65/35	1	25	230
Mitomycin C	C18 Zorbax, 4.6 × 250 mm	Acetonitrile/Water: 15/85	1.5	25	365

**Table 2:** Estimated loading efficiency of nanoparticles for Pac, Doc, and mitomycin C by HPLC analysis.

Initial Feeding			Drug in Pellet (μM)	Encapsulation Efficiency (*w*/*w* %)
	Drug (μM)	Ca (mM)		
Doc	100	7	2.01 ± 0.40	1.32 ± 0.21
Doc	100	0	0.69 ± 0.11	
Pac	100	7	27.75 ± 2.36	20.71 ± 4.34
Pac	100	0	7.04 ± 0.73	
Mito	100	7	0	0
Mito	100	0	0	

**Table 3:** Enhancement in cytotoxicity (%) of CA complexed drug vs. free drug on 4T1 and MCF-7 cells.

Drug Concentration	MCF-7		4T1	
	Pac	Doc	Pac	Doc
1 nM	−9.25 ± 0.79	14.98 ± 4.34		13.87 ± 3.69
10 nM	5.57 ± 1.22	10.31 ± 0.93	5.26 ± 1.11	10.43 ± 4.12
100 nM	15.87 ± 3.54	14.58 ± 5.05	5.64 ± 0.32	10.96 ± 2.32
1 µM	24.14 ± 2.21	14.05 ± 3.64	−6.06 ± 1.54	17.04 ± 2.12
10 µM			9.79 ± 3.78	

## Supplementary Materials

The following are available online at http://www.mdpi.com/2305-6304/6/1/12/s1. Figure S1: Apatite/drug (0 to 100 µM Pac) formed in bicarbonated media with 10:6 mM of total Ca:Pi in pH 6.5, 7.5 and 8.5, Figure S2: Standard curve for different concentrations of free drugs, Figure S3: MCF-7 Cells were seeded on 24 well plate at 5 × 104 cell/mL. After 24 h cells were treated with media only as control or apatite formulated with 1–7 mM calcium.

Supplementary File 1
