# Supplementary material for: Cytotoxicity Enhancement in Breast Cancer Cells with Carbonate Apatite-Facilitated Intracellular Delivery of Anti-Cancer Drugs
Source: Toxics. 2018 Feb 5;6(1):12. doi: 10.3390/toxics6010012 (PMC5874785; doi:10.3390/toxics6010012)
Supplement: Supplementary File 1 [file toxics-06-00012-s001.pdf]

Supplementary Materials: Cytotoxicity Enhancement in Breast Cancer Cells with Carbonate Apatite-Facilitated Intracellular Delivery of Anti-Cancer Drugs

Tahereh Fatemian and Ezharul Hoque Chowdhury

With fixed concentrations of Ca and Pi optimized for each pH (7:4.2 mM, 5:3 mM and 3:1.8 mM for pH 6.5, 7.5 and 8.5 respectively), effect of increasing drug concentration (20, 40, 60, 80 and 100 mM) on particle formation was evaluated (Figure S1).

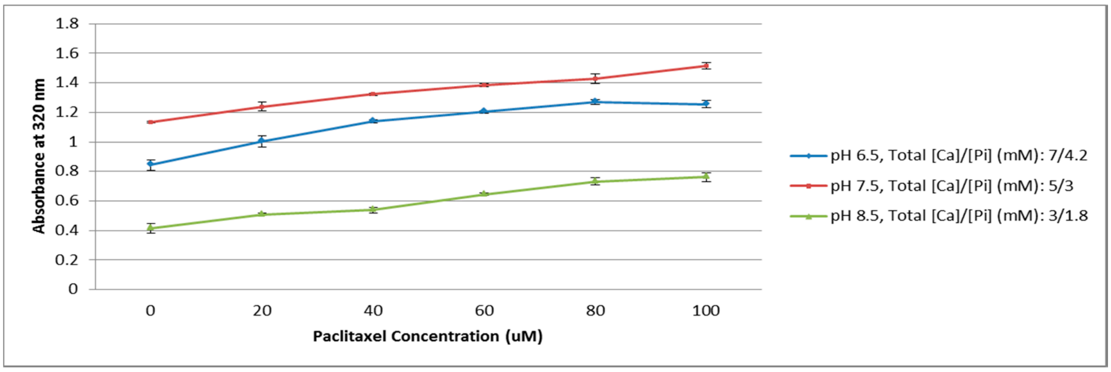

Figure S1. Apatite/drug (0 to 100 µM Pac) formed in bicarbonated media with 10:6 mM of total Ca:Pi in pH 6.5, 7.5 and 8.5. After 30 min incubation at 37 °C, turbidity of apatite/drug and free drug was measured at 320 nm against fresh media as blank. Values for apatite were calculated by deducting values for free drugs at different concentration. All experiments were done in duplicate and given as mean ± SD.

The standard curve of each drug was used to quantify the detected drug in supernatants and pellets (Figure S2).

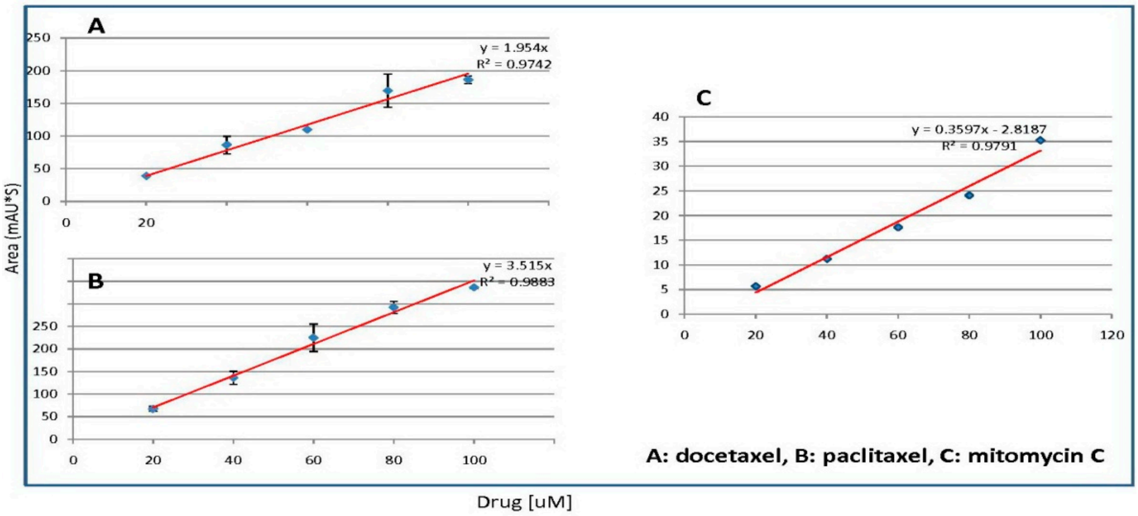

Figure S2. Standard curve for different concentrations of free drugs.

For in vitro studies, firstly the effect of nanoparticles formulated with different calcium concentrations was examined on MCF-7 cell viability (Figure S3).

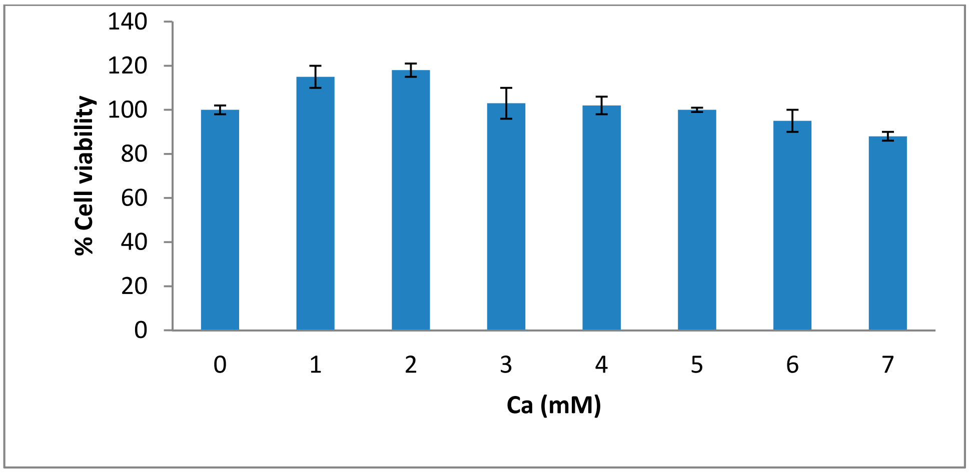

Figure S3. MCF-7 Cells were seeded on 24 well plate at 5 × 10^4 cell/mL. After 24 h cells were treated with media only as control or apatite formulated with 1–7 mM calcium. After 44 h of treatment cell viability was measured by MTT assay. Values are represented as % of cell viability as mean ± SD.
